# Relatedness and the evolution of mechanisms to divide labor in microorganisms

**DOI:** 10.1002/ece3.8067

**Published:** 2021-10-08

**Authors:** Ming Liu, Stuart Andrew West, Guy Alexander Cooper

**Affiliations:** ^1^ Department of Zoology University of Oxford Oxford UK; ^2^ St. John’s College Oxford UK

**Keywords:** cellular differentiation, coordination, division of labor, evolutionary theory, phenotypic noise, random specialization, signaling, social microbes

## Abstract

Division of labor occurs when cooperating individuals specialize to perform different tasks. In bacteria and other microorganisms, some species divide labor by random specialization, where an individual's role is determined by random fluctuations in biochemical reactions within the cell. Other species divide labor by coordinating across individuals to determine which cells will perform which task, using mechanisms such as between‐cell signaling. However, previous theory, examining the evolution of mechanisms to divide labor between reproductives and sterile helpers, has only considered clonal populations, where there is no potential for conflict between individuals. We used a mixture of analytical and simulation models to examine nonclonal populations and found that: (a) intermediate levels of coordination can be favored, between the extreme of no coordination (random) and full coordination; (b) as relatedness decreases, coordinated division of labor is less likely to be favored. Our results can help explain why coordinated division of labor is relatively rare in bacteria, where groups may frequently be nonclonal.

## Graphical abstract

Populations of social microorganisms use various mechanisms to divide labor over reproductive investment. We use mathematical models and simulations to show that relatedness has several impacts on selection for the mechanism to divide labor. Specifically, when relatedness is decreased, (a) random specialization becomes more likely to be favored than coordinated specialization, and (b) intermediate levels of coordination can arise. Our results help to explain why coordinated division of labor is relatively rare in bacteria, where groups may frequently be nonclonal.

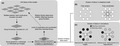

## 1 INTRODUCTION

Division of labor occurs when cooperating individuals specialize to carry out different tasks, to the benefit of all individuals involved (West & Cooper, 2016). Division of labor plays a key role across the tree of life: RNAs or replicators serving different functions form a genome together; cells specialize to perform different functions in multicellular organisms; and multicellular organisms perform different tasks in eusocial societies (Hart & Ratnieks, 2001; Higgs & Lehman, 2015; Hölldobler & Wilson, 1990; Ispolatov et al., 2012; Levin & West, 2017; Michod, 2005; Oster & Wilson, 1978; Simpson, 2012; Szathmáry & Smith, 1995).

Different species use different mechanisms to divide labor (Ackermann, 2015; Anderson et al., 2008; Schwander et al., 2010; Wahl, 2002; West & Cooper, 2016). In some bacteria species, individual cells specialize into distinct roles randomly and independently of one another by amplifying random fluctuations in the biochemical reactions of each cell (phenotypic noise). For example, whether or not a cell produces and secretes protease in *Bacillus subtilis* is determined randomly (Marlow et al., 2014; Veening, Igoshin, et al., 2008). Other microbial species use some form of coordination across cells when dividing labor. For example, cyanobacteria cells use signaling molecules to help determine which cells will develop into sterile, nitrogen fixing heterocysts (Herrero et al., 2016; Zhang et al., 2006). Finally, some insect species have a genetic component to division of labor, where an individual's role can depend on its genotype. For example, in some ants, different lineages develop into queens and workers at different rates (Hughes & Boomsma, 2008; Linksvayer, 2006; Schwander et al., 2010; Smith et al., 2008). These mechanisms are not mutually exclusive; for example, the ants using genetic determination also use coordination and signaling to control the proportion of different castes in the colony (Anderson et al., 2008; Schwander et al., 2010; Smith et al., 2008).

We lack evolutionary theory to explain why different species use different mechanisms to divide labor (Wahl, 2002; Cooper et al., 2020). Bacteria and other microorganisms provide a good system for tackling this problem, because both random and coordinated division of labor have evolved in these systems (Cooper et al., 2020). As a first step toward explaining this variation in microbes, Cooper et al. showed that coordination is more likely to be favored when group size is small and cooperation is more essential. Random specialization leads to variance in the proportion of helpers in a group, so some groups will end up with suboptimal proportions of helpers. The expected deviation from the optimal proportions of helpers is greater in smaller groups and leads to a larger fitness cost when cooperation is more essential.

However, this previous theory assumed clonal populations, with no conflict within groups (relatedness, *R* = 1). In contrast to this assumption, many microbial species appear to be in nonclonal populations where social interactions do not just take place with the same lineage (clone‐mate; i.e., *R* < 1). Indeed, at the scale of social interactions, such as within bacterial biofilms, populations often contain multiple species, let alone different lineages of the same species (Dragoš et al., 2018; Kim et al., 2016; Schiessl et al., 2019). Evidence for nonclonality (*R* < 1) in microbial social interactions has come from a variety of different experimental and observational studies on cooperation, both within and across species, as well as from the diversity of mechanisms that bacteria have evolved for attacking nonrelatives (Belcher et al., unpublished data; Bruce et al., 2017; Butaitė et al., 2017; Cordero et al., 2012; Fiegna & Velicer, 2005; Fisher et al., 2013; Foster et al., 2002; Gilbert et al., 2007; Granato et al., 2019; Hawlena et al., 2012; Simonet & McNally, 2021).

Theory has already shown that a lower relatedness leads to division of labor being less likely to be favored, or have a lower proportion of helpers (Cooper & West, 2018; Johnstone, 2000; Madgwick et al., 2018). In contrast, we do not know how nonclonality influences selection on the mechanism used to divide labor. For example, does nonclonality lead to a conflict of interest that disrupts coordination, and hence favors random specialization? Given that many cooperative microorganisms are nonclonal (*R* < 1), answering this question could help explain why coordinated specialization has been found relatively rarely in bacteria (Ackermann, 2015; West & Cooper, 2016).

We used a mixture of analytical and simulation approaches to examine the evolution of division of labor in both clonal and nonclonal populations. We focused on reproductive division of labor, where more cooperative “helpers” gain indirect fitness benefits by the aid they provide to less cooperative “reproductives.” Reproductive division of labor has been found in bacteria, algae, fungi, and slime molds; the cooperative traits in this form of division of labor include the following: fruiting body formation; nitrogen fixation; extracellular polysaccharide matrix production; beating flagella formation; programmed cell death; antibiotic production; adopting a tubular mitochondria; triggering an inflammatory response to eliminate competing bacteria; and releasing toxins (Table 1). We modeled cooperation as the production of a “public good” that benefits all the members of the group, because this form of cooperation is common in bacteria and other microbes (West et al., 2006, 2007). We asked whether division of labor was favored, and whether it was favored by random or coordinate specialization. We developed a relatively simple analytical model that compared the extreme cases of fully random and fully (perfectly) coordinated specialization. This model allowed us to examine analytically how different factors would influence selection for coordinated as opposed to random specialization.

We then developed a simulation model that allowed us to relax some assumptions of our analytical model. We allowed individuals to vary on a continuum, from purely random to fully coordinated, so that there could be intermediate levels of coordination. This simulation model allowed us to examine both: how any coordination could initially evolve from no coordination; and whether intermediate levels of coordination could be favored. In both our analytical and simulation models, we examined the influence of group size, the extent to which cooperation was essential, and within‐group relatedness.

**TABLE 1:** Microbial examples of reproductive division of labour, where some individuals specialised to tasks at the cost of their own fitness while the benefit of specialisation is shared by other conspecific individuals. Note that we have not included the examples of non‐reproductive division of labour (or mutual division of labour)

Specialisation	Species	Description	References
*Group 1: Sterile helpers*			
Fruiting body formation	*Myxococcus xanthus*	Sterile rods+ reproductive spores	Kaiser (2004), Fiegna and Velicer (2005), Konovalova et al. (2010), Higgs et al. (2014)
	*Dictyostelium discoideum*	Sterile stalk+ reproductive spores	Foster et al. (2002), Gilbert et al. (2007), Strassmann and Queller (2011), Madgwick et al. (2018), Dhakshinamoorthy and Singh (2021)
	*Bacillus subtilis* ^a^	Stalk‐like aerial structure+ spores	Branda et al. (2001)
Nitrogen fixing	Filamentous cyanobacteria, e.g., *Anabaena cylindrica*	Sterile heterocyst+ vegetative cell	Adams (2000), Zhang et al. (2006), Rossetti et al. (2010), Herrero et al. (2016)
Beating flagella	*Volvex cateri*	Soma cell with flagella+ non‐flagellated germ cells	Kirk (2005), Herron and Michod (2008)
	*Volvox* (others)	Soma cell with flagella+ non‐flagellated germ cells	Shelton et al. (2012), Matt and Umen (2016)
Bacteriocin production	*Escherichia coli*	Colicin producing cells (3%) and others. Mechanism involves LexA repressor and SOS regulation. The producer is lysed (killed) during colicin release	Mulec et al. (2003), Cascales et al. (2007), Kamenšek et al. (2010), Mader et al. (2015), Mavridou et al. (2018)
	*Pseudomonas aeruginosa*	Some individuals heterogeneously synthesise and release pyocin through cell lysis (death)	Michel‐Briand and Baysse (2002), Waite and Curtis (2009), Mei et al. (2021)
	*Xenorhabdus bovienii* and *Xenorhabdus koppenhoeferi*	Different bacteriocins are produced by different colonies and intraspecific inhibition is stronger than interspecific inhibition. The bacteriocins are similar to pyocin	Hawlena et al. (2012)
Triggering host inflammation response	*Salmonella enterica*	Some individuals heterogeneously express secretion system and sacrifice themselves to eliminate competitors	Ackermann et al. (2008), Sturm et al. (2011), Diard et al. (2013), Bumann and Cunrath (2017)
Virulence	*Cryptococcus gattii*	Some individuals adopt a tubular mitochondrion to facilitate others' growth	Voelz et al. (2014), Farrer et al. (2018)
Apoptosis	*Saccharomyces cerevisiae*	Some individuals undergo apoptosis after multicellular cluster formation.	Ratcliff et al. (2012)
*Group 2: Non‐sterile helpers*			
Degrading extracellular substances/ nutrients	*Pseudoalteromonas *sp. strain S91	Differential production in chitinolytic enzymes	Baty et al. (2000)
Antibiotic production	*Vibrionaceae* isolates	Genetic analyses show that within populations, broad‐range antibiotics are produced by few genotypes, whereas all others are resistant, suggesting cooperation between conspecifics	Cordero et al. (2012)
	*Streptomyces coelicolor*	Bistable switch for antibiotic‐producing phenotype	Mehra et al. (2008)
	*Streptomyces coelicolor*	Amplification and deletions to the chromosome causes differences in antibiotic production	Zhang et al. (2020)
Biofilm formation	*Bacillus subtilis*	Matrix‐producing cell type and others (bistable switch).	Branda et al. (2004), Branda et al. (2006), Dubnau and Losick (2006), Chai et al. (2008), Veening et al. (2008a), López and Kolter (2010), Marvasi et al. (2010), Marlow et al. (2014), Dragoš et al. (2018)
	*Bacillus subtilis*	Exoprotease‐producing cell type and others	Veening et al. (2008a), Marlow et al. (2014)
	*Candida auris* (yeast pathogen)	Aggregative and non‐aggregative phenotypes.	Brown et al. (2020)
Mobility chemical production	*Pseudomonas fluorescens*	One of the two strains produces wetting polymer.	Kim et al. (2016)

^a^ Cell types include motility, surfactin production, matrix production, protease production, and sporulation (Vlamakis et al., 2013; van Gestel et al., 2015).

## 2 METHODS AND RESULTS

### 2.1 Analytical game theory model

#### 2.1.1 Population structure and life cycle

We employed a deliberately simple model, focusing on factors of general importance across many microbial species, rather than a specific model for a particular species. We assumed that social groups are formed by *l* founding cells, each of which spawns a lineage with *m* cells, giving a final group size of n=lm (Haystack model; Maynard Smith (1964). Hence, the whole‐group relatedness is 1/l (including self; Pepper (2000).

When the group has reached a size of *n*, division of labor may occur with individuals becoming either a sterile helper or a pure reproductive. The reproductives then produce a large number of offspring. Although we are interested in all forms of reproductive division of labor, we assume the extreme case of sterile helpers and pure reproductives in the model for mathematical tractability. Across different microbial species, there are examples of reproductive division of labor with sterile helpers, and with helpers that just show reduced reproduction (Table 1). After reproduction, individuals in the current generation all die (nonoverlapping generations), and all offspring disperse globally and compete to found groups in the next iteration of the group life cycle (global competition). The fecundity of each reproductive depends upon the level of cooperation in the group, which is determined by the proportion of helpers in the group.

We assumed that cooperation takes the form of producing a public good, which is shared with all members of this group. Bacteria and other microorganisms produce a large range of public goods, including iron scavenging siderophore molecules, and enzymes to digest proteins, aromatic compounds, or antibiotics (Diggle et al., 2007; Frost et al., 2018; Griffin et al., 2004; West et al., 2007). Public good models have been very useful for examining both cooperation and division of labor in microbes (Cooper & West, 2018; Frank, 2010; Lee et al., 2016; Sasaki & Uchida, 2013; West & Buckling, 2003). Other forms of cooperation such as beating flagella to keep a group of cells afloat can also be thought of as a form of public good cooperation (Herron & Michod, 2008; Michod, 2006). Another form of cooperation that involves division of labor is when cells sacrifice themselves to produce bacteriocin toxins that kill unrelated cells—this can be conceptualized as harming nonrelatives to help relatives, by reducing competition for resources, which is also a form of public good cooperation, directed at relatives (Gardner et al., 2004; Granato et al., 2019).

#### 2.1.2 Reproductive division of labor

We assumed that individuals in the last generation of the group life cycle develop into either pure reproductives or sterile helpers. Sterile helpers do not reproduce, but provide cooperative benefits that linearly increase the fecundity of pure reproductives in the group. Specifically, when a proportion *P* of individuals in the group are sterile helpers, the fecundity of a reproductive is proportional to: 1−ϵ+ϵP, where 0 ≤ *ϵ* ≤ 1. Here, 1 − *ϵ* represents the baseline fecundity, in the absence of any helpers, and *ϵP* represents the increase in fecundity gained from the cooperative behavior of sterile helpers. The parameter *ϵ*, which we call the essentiality of cooperation, measures the degree to which reproductive fecundity is dependent on the amount of help in the group.

#### 2.1.3 The evolving target proportion of helpers (*q*)

Whether an individual becomes a sterile helper or a pure reproductive depends on a heritable trait and its mechanism for dividing labor. We assumed that there is an evolving trait, the target proportion of helpers, *q*, that specifies the proportion of helpers in the group that the individuals aim to achieve. This is akin to the optimal proportion of helpers for the lineage's founder. The target proportion of helpers, *q*, is a genotypic property of a focal individual, whereas the realized proportion of helpers in the group, *P*, is a phenotypic property of the whole group (depending upon the mechanism used to coordinate labor).

#### 2.1.4 Mechanisms for dividing labor

**FIGURE 1:**
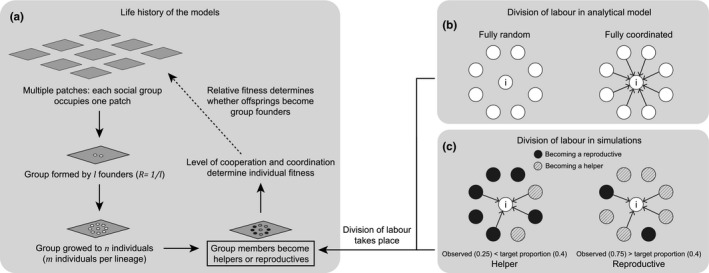
Life history assumptions. (a) We outline the life history assumptions common to both our analytical and simulation model. Parts b and c show the mechanisms by which division of labor occurs in our (b) analytical and (c) simulation models, respectively. In our analytical model (b), there are two mechanisms to divide labor: fully random specialization, where no communication is involved and each individual decides its phenotype probabilistically according to its target proportion of helper (a heritable trait, *q*), and fully coordinated specialization, where individuals assess the intended phenotype of all other members of the same lineage and adjust their developmental plan to match their target proportions of helpers. In our simulation model (c), the level of coordination is continuous, going from fully random to fully coordinated. We varied the level of coordination (*s*) by varying the proportion of the group that each individual signals and coordinates with. In the examples shown, we have assumed that the target proportion is 0.4, and so the focal individual would adopt a helper role in the left case and a reproductive role in the right case

We examined two different mechanisms for dividing labor: fully random specialization and fully coordinated specialization. With random specialization, each individual becomes a helper with probability equal to its target proportion of helpers (Figure 1a; i.e., *P* = *q* or *P* ≠ *q*). In contrast, with coordinated specialization, individuals use within‐lineage signaling to produce lineages with an exact proportion of *q* helpers (Figure 1b; i.e., *P* = *q*). We assumed that coordinators pay a relative cost of sending and receiving signals (*θ*).

#### 2.1.5 Mathematical analysis

We carried out a two‐step invasion analysis. First, we calculated the evolutionary stable target proportion of helper for each mechanism, in uniform populations where every individual uses the same mechanism to divide labor (Section S1.2–S1.4). Second, we then examined when a mutant strain with the alternative mechanism for dividing labor could invade the population.

#### 2.1.6 The relative fitness of random specializers

Let us assume that a focal founder employs fully random specialization with a target proportion of helpers, *q*, in a group that otherwise has a target proportion of helpers, *Q*. In this case, the expected fitness of the focal founder can be written as (Section S1.2):

(1) $$w_{\mathrm{FR}}\left(q,Q\right)=\left(1‐q\right)\left(1‐\epsilon +\epsilon q/l+\epsilon \left(l‐1\right)Q/l‐\epsilon q/lm\right).$$

The first term in parentheses is the expected proportion of reproductives in the founder's lineage (1 − *q*). The second term in parentheses is the expected fecundity of each reproductive, which is determined by: the baseline fecundity, 1 – *ϵ*; the expected increase in fecundity from within‐lineage help, ϵq/l; the expected increase in fecundity from across‐lineage help, ϵ(l−1)Q/l; and the final term, *ϵq*/*lm*, is the expected fecundity cost due to random deviations from the target proportion of helpers. In particular, it does not matter whether the other founders use random or coordinated specialization because the expected levels of helping across the social group (i.e., *Q*) are the same for focal mutant lineage in either case.

We calculate the Evolutionarily Stable Strategy (ESS) for the target proportion of helpers ($q_{\mathrm{FR}}^{*}$), which is the target proportion of helpers that cannot be invaded by any other strategy in a population of random specializers (Section S1.3 (Maynard Smith & Price, 1973)). This gives:

(2) $$q_{\mathrm{FR}}^{*}=\left\{\begin{array}{cc} 0, & \text{if}\;\epsilon <lm/\left(l\left(m+1\right)‐1\right)\\ 1‐\frac{lm‐\epsilon }{\epsilon \left(lm+m‐2\right)}, & \text{otherwise} \end{array}\right.$$

More essential cooperation (higher *ϵ*), smaller lineage sizes (smaller *m*), and fewer lineages (smaller *l*) lead to a higher level of cooperation in random groups (larger $q_{\mathrm{FR}}^{*}$) (Section S1.3).

#### 2.1.7 The relative fitness of coordinated specializers

We now assume that the focal founder employs fully coordinated specialization to produce an exact proportion *q* of helpers within its lineage and that the other founders have an exact target proportion of helpers, *Q*. We assume that cells do not coordinate with cells from other lineages. We have the expected fitness as:

(3) $$W_{\mathrm{FC}}\left(q,Q\right)=\left(1‐\theta \right)\left(1‐q\right)\left(1‐\epsilon +\epsilon q/l+\epsilon \left(l‐1\right)Q/l\right).$$

The first term in parentheses captures the cost of coordination, the second term is the proportion of reproductives in the focal lineage, and the final term is the expected fecundity of each reproductive in the focal lineage (in the absence of coordination costs). This last term is equal to the expected fecundity of random specializers without the cost to random specialization (second last term of Equation (1); ϵ(l−1)Q/l). Once again, it does not matter in Equation (3) whether the other (nonfocal) group founders employ coordination or random specialization when dividing labor (see also Section S1.2).

We can calculate the ESS target proportion of helpers in a population of coordinators, giving:

(4) $$q_{\mathrm{FC}}^{*}=\left\{\begin{array}{cc} 0 & \text{if}\;\epsilon </\left(l+1\right)\\ 1‐\frac{l}{\epsilon \left(l+1\right),} & \text{otherwise} \end{array}\right.$$

Again, we find that more essential cooperation (higher *ϵ*) and fewer lineages (lower *l*), favor a higher level of cooperation (larger $q_{\mathrm{FC}}^{*}$) (Section S1.4). In contrast, because coordinated specializers produce a deterministic proportion of helpers, the ESS level of coordinated cooperation does not depend on the size of the lineage (*m*) The level of cooperation in a population of coordinators is greater than the level of cooperation in a population of random specializers ($q_{C}^{*}>q_{R}^{*}$; Section S1.5).

#### 2.1.8 Invasion analysis

We now determine the conditions in which each mechanism is either stable or invadable by the other mechanism. A focal mutant employing random specialization can invade a population of coordinated specializers if $w_{\mathrm{FR}}\left(q_{\mathrm{FC}}^{*},q_{\mathrm{FC}}^{*}\right)>w_{\mathrm{FC}}\left(q_{\mathrm{FC}}^{*},q_{\mathrm{FC}}^{*}\right)$, giving the condition:

(5) $$\epsilon <\frac{\theta lm}{\theta lm\left(1‐q_{\mathrm{FC}}^{*}\right)+q_{\mathrm{FC}}^{*}}$$

Alternatively, a focal mutant employing coordinated specialization can invade a population of random specializers if $w_{\mathrm{FC}}\left(q_{\mathrm{FR}}^{*},q_{\mathrm{FR}}^{*}\right)>w_{\mathrm{FR}}\left(q_{\mathrm{FR}}^{*},q_{\mathrm{FR}}^{*}\right)$, giving the condition:

(6) $$\epsilon >\frac{\theta lm}{\theta lm\left(1‐q_{\mathrm{FR}}^{*}\right)+q_{\mathrm{FR}}^{*}}$$

If the cost of coordination (*θ*), the number of lineages (*l*), and the final size of lineages (*m*) is sufficiently large (*θlm* ≥ 1), then random specialization can always invade and is always stable to invasion. Otherwise (if *θlm* < 1), more essential cooperation (larger *ϵ*), less costly coordination (lower *θ*), fewer lineages (lower *l*), smaller lineage sizes (lower *m*), and a higher level of cooperation (larger $q_{c}^{*}$ or $q_{R}^{*}$) favor the invasion of coordinated specialization.

In the above, we assumed that the level of cooperation of an invading mutant is equal to the ESS for the resident population, so that the only difference between competing mechanisms is the way that helpers are produced and not the relative target proportion of helpers. We find the same qualitative results when mutants employ the optimal target proportion of helpers for their mechanism to divide labor (coordinated division favors a higher target proportion of helpers; modeling details are provided in Section S2). We also modeled the invasion under various costs of coordination and found coordinated specialization is more dominant when the cost is lower (Section S1.7).

### 2.2 Analytical predictions

**FIGURE 2:**
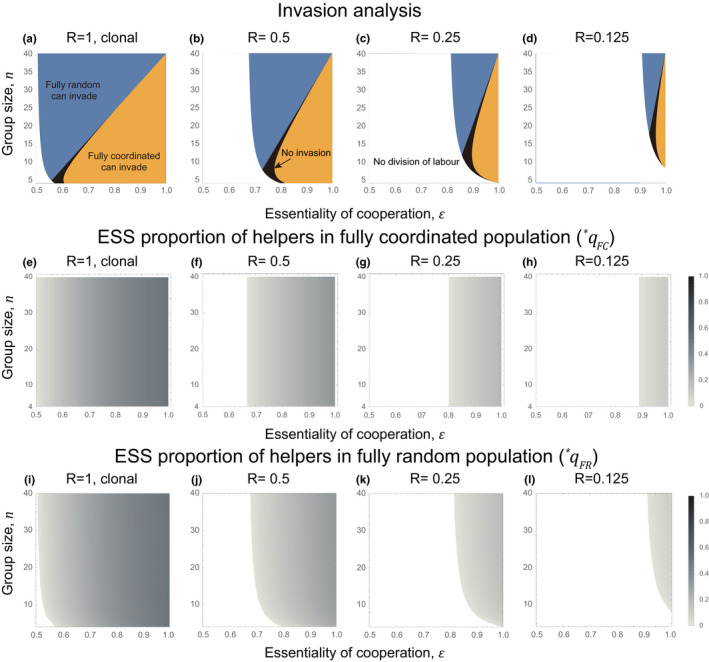
Invasion analysis and evolutionary stable strategy (ESS) in the analytical model. We performed invasion analyses to see if fully coordinated specializers can invade fully random specializers, and vice versa. We fixed the cost of coordination (*θ* = 0.025) in all panels and varied the number of founders (*l* = 1, 2, 4, 8) to change the relatedness of populations. The different colors represent areas of parameter spaces where fully random specializer can invade fully coordinated population (blue), fully coordinated specializer can invade fully random population (orange), no invasion (black), and no division of labor (white). The *x*‐axis is the essentiality of cooperation (*ϵ*) and the *y*‐axis is the size of social group (*n* = *lm*). The different panels show: (a–d) the results of the invasion analysis; (e–h) the ESS proportion of helpers in populations with fully coordinated specializers ($q_{\mathrm{FC}}^{*}$); (i–l) the ESS proportion of helpers in populations with fully random specializers ($q_{\mathrm{FR}}^{*}$)

Our predictions are consistent with previous theory where groups were assumed to be clonal (Figure 2a, e, i; Cooper et al., 2020). When cooperation is more essential (higher *ϵ*), there is a larger opportunity cost from producing suboptimal proportions of helpers and so random specialization is disfavored. When the size of the lineage increases (higher *m*), the relative variance in the proportion of helpers produced by random specialization decreases, which leads to a smaller cost of stochasticity (Figure 2e,i; see also Section S1.6).

Moving from clonal groups (*l* = 1) to nonclonal groups (*l* > 1), we found that as relatedness decreased (higher *l*): (a) division of labor is less likely to be favored (Figure 2, left to right columns); and (b) a lower proportion of helpers is favored (Figure 2, left to right columns). Consistent with previous theory, these patterns reflect the smaller inclusive fitness benefit of altruism (sterile helping) toward individuals that are less likely to be kin (Section S1.3 and S1.4) (Cooper & West, 2018; Hamilton, 1964a, 1964b; Johnstone, 2000; Reeve et al., 1998; Reeve & Shen, 2006).

We found that groups with a lower relatedness (more lineages; higher *l*) favor the evolution of random specialization over coordinated specialization (Figure 2). When there are more lineages, the variance in the proportion of helpers produced by a focal lineage has less of an impact on the total proportion of helpers across the group. This diminishes the expected cost of stochasticity to a focal founder employing random specialization. This result differs from previous theory, which had only considered the case of clonal groups (*R* = 1).

We also found that, as relatedness decreases, there is an increase in the size of the intermediate area where neither mechanism could invade the other (Figure 2); in our alternate analysis, there is an increase in the intermediate area where the two mechanisms could mutually invade the other (Figure 6; Section S2). By construction, our analytical models only allow for the evolution of fully coordinated or random specialization. We hypothesized that this expanding intermediate region corresponds to where partial coordination could be favored. To test this hypothesis, we next developed individual‐based simulations in which intermediate mechanisms could evolve.

### 2.3 Individual‐based simulations

Our analytical model examined the extreme cases where division of labor was either completely random, or fully coordinated. In order to investigate the continuum between these two cases, and to determine whether coordination can “gradually” evolve, we developed an individual‐based simulation.

#### 2.3.1 The evolving level of coordination (*s*)

We assumed that the extent to which individuals adjust their phenotype depending upon the phenotype of their group mates can vary continuously, as defined by their probability, *s*, of being “coordinated” with each group neighbor. If division of labor is at least partially coordinated (*s* > 0), an individual may interact with some group neighbors, via signals or cues, and can take account of these neighbors’ intended phenotype when specializing. If *s* = 0, then an individual is not coordinated with any neighbors and we assume that the individual is a fully random specializer, with helper probability equal to its target proportion of helpers (*q*).

More specifically, the focal individual has probability *s* of establishing a one‐way link with each group member and receives information about the neighbor's intended phenotype (arrows in Figure 1c). We modeled the signaling process in this way because in many systems the spatial arrangements of individuals remain relatively static when division of labor takes place (van Gestel et al., 2015; Keller & Surette, 2006; Yanni et al., 2020). Consequently, one way of thinking about the coordination parameter *s* is that it measures the relative proportion of the group that a focal individual is close enough to coordinate with.

We assumed the initial phenotype of all individuals is reproductive, as many organisms would start as newly divided cells but then specialize to become helpers later (Ackermann et al., 2008; Herrero et al., 2016). After the coordination network is formed, the individuals within the group are randomly sampled, one individual at a time, to determine whether it changes its intended phenotype. The type‐changing decision depends on comparing its target proportion of helpers with the observed proportion of “intended helpers” amongst all the cells that it interacts with. If the observed ratio is larger than target proportion, the sampled individual sets its developmental plan to become an “intended reproductive,” and vice versa (Figure 1c).

We designed the metabolic cost of coordination as a function of the level of coordination, cost(*s*) = *θ*(1 − *e* ^−5^ *^s^*), where *θ*, the cost coefficient, has the same value as in analytical model. This cost of coordination is nonlinear, with a decelerating slope (i.e., saturating with increasing *s*; $\frac{d^{2}\left(\theta \left(1‐e^{‐5s}\right)\right)}{ds^{2}}<0$) (Foster, 2004). The high initial cost can be thought of as the cost of building the coordination machinery (e.g., protein interaction networks), and then, increased coordination improves the efficiency of that machinery (Crespi, 2001; Wilkinson, 1988). In addition, the cost function has the same or very similar cost as the analytical model when *s* = 0,1 to make the modeling results more comparable.

#### 2.3.2 Fitness calculation and the coevolving traits

We assumed that the fecundity of an individual, *w*, is given by:

(7) $$w=\left(1‐\theta \left(1‐e^{‐5s}\right)\right)\left(1‐h\right)\left(1‐\epsilon +\epsilon P\right)$$

The first term in parentheses contains the cost of coordination; the second term specifies the individual's phenotype, where *h* is 1 for a helper and 0 for a reproductive. The third term captures the benefits of group cooperation, which is higher when there is a larger proportion of helpers. Note that the value of *h* for each individual is determined by both the target proportions of helpers and the level of coordination, and the two traits are coevolving in the simulations.

#### 2.3.3 Mutation of the coevolving traits

We assumed the traits can mutate between generations. For each trait of a new founder, the mutation rate is *p*_mut_ = 0.01. If a mutation occurs, we perturb the parental trait value by a normally distributed deviation with mean of 0 and standard deviation of 0.1, constrained at the boundaries [0,1].

#### 2.3.4 Analysis of the simulations

For each combination of parameters, we investigated two scenarios: (a) fully random specialization (*s* = 0), where only the target proportion of helpers (*q*) was allowed to evolve; (b) both the target proportion of helpers (*q*) and the level of coordination (*s*) were allowed to evolve. We used the case of fully random specialization (*s* = 0) as a “control” to examine the influence of coordination. In all cases, we repeated the simulation 10 times, ran 10^5^ generations for each simulation, and let division of labor take place when the population size is around 10^4^ individuals (i.e., $lm\left[10^{4}/lm\right]$) to ensure that the trait values converged to their evolutionary equilibria.

### 2.4 Simulation Results

#### 2.4.1 Intermediate coordination coevolved from simulations

Examining dynamics over time, we found that intermediate coordination can evolve from either fully random specialization or fully coordinated specialization (Figure 4). The level of coordination favored can be intermediate between the extremes of full (*s* = 1) or no (*s* = 0) coordination. Comparing across different runs of the simulation, the level of coordination (${\bar{S}}_{\text{coev}}$) showed greater variation than the proportion of helpers (${\bar{P}}_{\text{coev}}$) (Figure 4; gray lines are significantly more variable than pink lines; *F* test, *p* < 10^−5^). Greater variation can be expected because the level of coordination has a smaller influence on fitness than the proportion of helpers, via its influence on the *θs* term where *θ* is much smaller than 0.1 in Equation (7) (weaker stabilizing selection).

**FIGURE 3:**
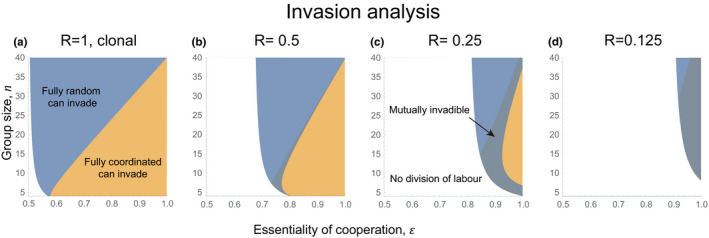
Invasion analysis of fully random or fully coordinated specializers when invaders can have different target proportion of helpers from the residents. We performed invasion analysis over various relatedness setting and see if fully coordinated specializers can invade fully random specializers, or vice versa. We fixed the cost of coordination (*θ* = 0.025) in all panels and vary the number of founders (*l* = 1, 2, 4, 8) to change the relatedness of populations. Colored areas represent parameter spaces where fully random specializer can invade fully coordinated population (blue), fully coordinated specializer can invade fully random population (orange), mutually invadable (brown), and no division of labor (white). The *x*‐axis is the essentiality of cooperation (*ϵ*) and the *y*‐axis is the size of social group (*n* = *lm*). See Section S2 for more details of this analytical model

**FIGURE 4:**
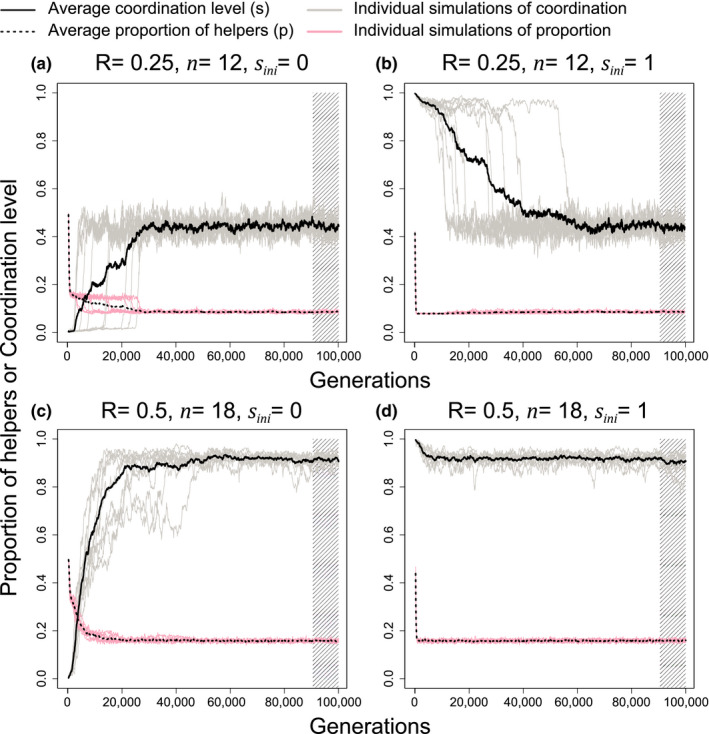
The coevolving dynamics of helper proportion and coordination level. Coordination is shown in gray and solid black line, while helper proportion is shown in pink and broken black lines. Each panel shows the dynamics of 10 repeated simulations and the essentiality of cooperation is set to 1.0. The panels show how some level of coordination can evolve from initially no coordination, and that there can be greater variance in the level of coordination, compared with the level of helping. Stripped areas are the generations used in plotting the heatmap (Figure 5). Note that group size (*n*) is equal to the number of lineages (*l*) times lineage size (*m*)

#### 2.4.2 Agreement with analytical models

**FIGURE 5:**
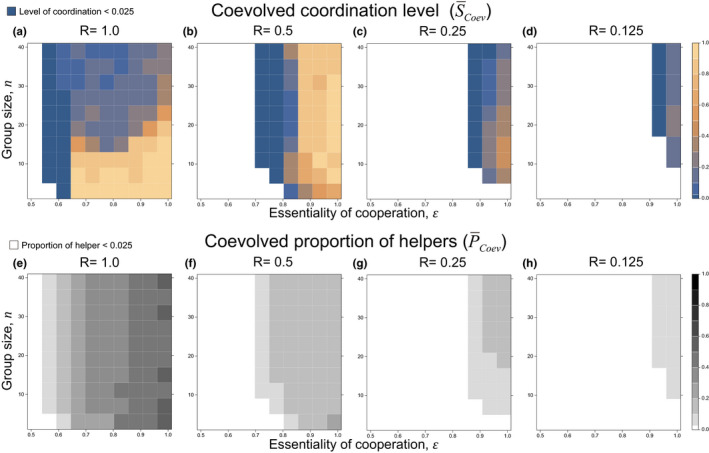
Coevolved level of coordination and proportion of helpers in the simulations. (a–d) The evolved coordination level plotted against the essentiality of cooperation (*x*‐axis; *ϵ*) and group size (*y*‐axis; *n* = *lm*). Each panel represents the results of different relatedness setting. The blueish colors represent the evolved mechanism of division of labor is closer to random specialization, whereas the orangish colors represent the evolved mechanism is closer to coordinated specialization. Each grid is an average of 10 repeated simulations where the average is taken from the last 10% of 10^5^ generations. (e–h) The evolved proportion of helpers. Darker shades mean there are more helpers in the population. Coordination levels when proportion of helpers is below 0.025 is not plotted as we regard it as no division of labor

**FIGURE 6:**
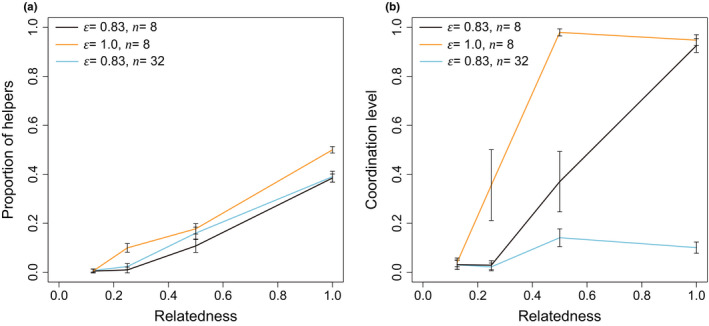
Effects of relatedness on: (a) proportion of helpers (*p*); and (b) level of coordination (*s*). Note the symbols in parameter settings represent essentiality (*ϵ*) and group size (*n* = *lm*). Each point is the average of the last 10% time‐steps of the 10^5^‐step simulations; error bar represents the standard error from 10 repetitions

Consistent with our analytical model, we found that as relatedness decreases: (a) division of labor is less likely to be favored (shaded area decreases as go across Figures 5e‐h and 6a); (b) a lower proportion of helpers is favored (lighter shading as go across Figures 5e‐h and 6a); (c) mechanism for dividing labor shifts from more coordinated to more random in general (Figures 5a‐d and 6b). In clonal groups, we also found coordinated specialization is more favored when essentiality is high and group size is small (Figures 5a and 6b).

#### 2.4.3 Precision of coordination

As relatedness decreases, we found that less precise coordination is favored—the brightest shading goes from orange to brownish blue across Figure 5a‐d. In other words, the maximal level of coordination is smaller when relatedness is low. This pattern may reflect that there are less helpers in low‐relatedness population, reducing the relative advantage of more precision division. The pattern may also reflect random specialization being a cheating strategy that exploits the costly coordination practiced by other lineages. The decreased maximal level of coordination along relatedness contrasts the analytical models as intermediate levels of coordination are not included in those models.

#### 2.4.4 Robustness of results

We confirmed the robustness of our conclusions with several additional simulations. Our simulation results were not changed when we varied the initial starting conditions (Section S3.3). We found the same qualitative pattern when the cost of coordination increased linearly or accelerating, rather than decelerating with the level of coordination (Section S3.5–S3.7). When analyzing purely random division, with no coordination, the results of our simulation were in close agreement with our analytical model (Section S3.1). If cells only coordinate within their own lineage, then lower levels of coordination are favored, because coordination is less able to reach the target proportion of helpers (Section S3.4). Quantitative differences between our analytical and simulation results appear to arise from our simulation allowing the proportion of helpers and the level of coordination to coevolve (Section S3.2).

## 3 DISCUSSION

We found that as relatedness decreased (lower *R*), there was reduced selection for division of labor to be coordinated. We first tackled this issue analytically, examining the extremes of division of labor by fully coordinated and fully random specialization. In these models, we found that when relatedness was lower (lower *R*), random specialization was more likely to be favored (Figures 2 and 3). We then developed a simulation model that allowed us to examine intermediate levels of coordination. Our simulation showed that when relatedness was lower (lower *R*), that lower levels of coordination were favored to divide labor (Figures 5 and 6). These results differ from previous theory, which had only considered the case of clonal groups (*R* = 1; Cooper et al., 2020).

Why did a lower relatedness lead to reduced selection for coordinated division of labor? One factor is that when relatedness is lower, lower levels of helping are favored (Figures 2 and 5), and so helping has a smaller influence on fitness (Equations 2 and 4). Consequently, there is weaker selection to coordinate division of labor precisely. Another factor is that paying a personal cost to coordinate division of labor can be seen as a form of cooperation. A lower level of coordination, or random division of labor, which avoids the personal cost of coordination, can hence be seen as form of cheating. When relatedness is lower, there is reduced selection for cooperation, and increased selection for cheating.

Our results can help explain the distribution of mechanisms to divide labor across bacteria and other microorganisms. Many microbe species appear to use random specialization to produce division of labor, based upon “phenotypic noise” (Ackermann, 2015; Dubnau & Losick, 2006; Lewis, 2010; Smits et al., 2006; Veening et al., 2008; West & Cooper, 2016). Further, many of these species are likely to interact in nonclonal populations (*R* < 1). For example, social bacteria *Myxococcus xanthus*, fungal pathogen *Cryptococcus gattii*, social amoeba *Dictyostelium discoideum*, and a range of species in the gut microbiome (Dragoš et al., 2018; Farrer et al., 2018; Fiegna & Velicer, 2005; Foster et al., 2002; Gilbert et al., 2007; Simonet & McNally, 2021; Voelz et al., 2014). We have shown that, in nonclonal populations, appreciable levels of coordination are only favored when cooperation is relatively essential (high *ϵ*; Figures 2 and 4). In contrast, the most striking examples of coordinated division of labor are in clonal populations, such as colonial green algae *Volvox carteri*, and filamentous cyanobacteria *Anabaena cylindrica* (Herrero et al., 2016; Kirk, 2001; Matt & Umen, 2016; Rossetti et al., 2010). Nonetheless, our conclusions here are speculative ‐ as data on more species becomes available, it would be extremely useful to carry out a formal across species test of our predictions.

Our models also supported the predictions of previous theory. We found that as relatedness decreases: (a) division of labor is less likely to be favored (Figures 2 and 5); (b) a smaller proportion of helpers is favored (Figures 2 and 5). In addition, we found that coordinated division of labor is more likely to be favored when cooperation is more essential, or group size is smaller (Figures 2 and 5). These results agreed with previous theory examining either division of labor (Ackermann et al., 2008; Cooper & West, 2018; Michod & Roze, 1999; Cooper et al., 2020), or reproductive skew (Johnstone, 2000; Reeve et al., 1998; Reeve & Shen, 2006). Empirically, both experimental and comparative studies have found a lower proportion of helpers when relatedness is lower (Fisher et al., 2013; Langer et al., 2004; Madgwick et al., 2018).

Finally, there are at last two important avenues for future progress in this area. Empirically, data are required on both the molecular machinery for dividing labor in a wider range of species, and the relatedness structure of natural populations (Hall et al., 2020; Madgwick et al., 2018; Olm et al., 2021; Simonet & McNally, 2021; Speed & Balding, 2015). Data on these and other ecological parameters, such as group size or the relative importance (essentiality) of cooperation, would allow us to look for broad across species patterns. Theoretically, we have developed a deliberately simple model that could be applied widely. It would be very useful to develop more specific models, based on the mechanisms of particular species. As well as allowing specific coordination mechanisms to be modeled, this would allow other factors to be investigated: (a) division of labor being adjusted in response to population density (Bumann & Cunrath, 2017; Maldonado‐Barragán & West, 2020; Mavridou et al., 2018); (b) strategies producing a “deceptive” coordination signal that could be selected for in low‐relatedness populations; (c) incorporating spatial structure to model case‐specific cooperative interactions; (d) phenotypes that can be changed in later life stages (Bergmiller & Ackermann, 2011; Strassmann & Queller, 2011); and (e) division of labor where all individuals are specialized to tasks not related to reproduction (Armbruster et al., 2019; van Gestel et al., 2015; Nikel et al., 2014).

## CONFLICT OF INTEREST

None declared.

## AUTHOR CONTRIBUTION

**Ming Liu:** Conceptualization (equal); Formal analysis (equal); Investigation (lead); Methodology (supporting); Validation (equal); Visualization (lead); Writing‐original draft (lead); Writing‐review & editing (equal). **Stuart Andrew West:** Conceptualization (equal); Formal analysis (equal); Funding acquisition (lead); Investigation (supporting); Methodology (supporting); Supervision (equal); Visualization (supporting); Writing‐original draft (supporting); Writing‐review & editing (equal). **Guy Alexander Cooper:** Conceptualization (equal); Formal analysis (equal); Investigation (supporting); Methodology (lead); Supervision (equal); Validation (equal); Visualization (supporting); Writing‐original draft (supporting); Writing‐review & editing (equal).

### OPEN RESEARCH BADGES

This article has earned an Open Data Badge for making publicly available the digitally‐shareable data necessary to reproduce the reported results. The data is available at https://github.com/mingpapilio/Codes_DOL_Mechanisms_Nonclonal.

## Supporting information

Supplementary Material

## Data Availability

All results are generated using Mathematica and C. The codes and generated data used for this study are available at: https://github.com/mingpapilio/Codes_DOL_Mechanisms_Nonclonal.
