# Supplementary material for: Relatedness and the evolution of mechanisms to divide labor in microorganisms
Source: Ecol Evol. 2021 Oct 8;11(21):14475–89. doi: 10.1002/ece3.8067 (PMC8571581; doi:10.1002/ece3.8067)
Supplement: Supplementary file 1 — Supplementary Material [file ECE3-11-14475-s001.docx]

**Supplementary Information**

**1. Further analyses of the analytical game theory model**

In this section, we provide more details for math derivations as well further analyses of the results. In section 1.1, we recall the model designs, in particular the life cycles, population structures, and social interactions of the analytical model. Section 1.2 shows the derivation of fitness of fully random specialisation. In sections 1.3-1.4, we derive and analyse the evolutionarily stable strategies (ESSs) of the target proportion of helpers for fully random and coordinated specialisation. Section 1.5 shows that the ESS target proportion of helpers for fully coordinated specialisers is always equal to or greater than the ESS target proportion of helpers for fully random specialisers. In section 1.6, we rearrange the fitness functions to compare the cost of the two specialising mechanisms. Section 1.7 illustrates the effect of the cost of coordination on the invasion conditions.

*1.1 Population, life cycle and social interactions*

We assume that the population is structured into distinct social groups that each start with $l$ founding cells, which synchronously replicate to form a lineage of cells in the group. The last generation of the group life cycle occurs when the group has reached the size of $lm$ cells ($l$ lineages, $m$ cells per lineage), at which point fecund cells produce a large number of offspring that disperse globally to found new groups. All cells from the previous groups then die (non-overlapping generations) and the life cycle begins again.

We assume that cells in the last generation of the group life cycle can become either sterile helpers that produce a public good for the group, or pure reproductives that do not produce a public good but use the good to produce a larger number of dispersing offspring. We assume that the fecundity of a reproductive is equal to $\left( 1-\epsilon\right)+\epsilon P$, where the parameter $\epsilon$ is the essentiality of cooperation, and the variable $P$ is the proportion of helpers produced in the group in the last generation.

Whether and how cells in the last generation become helpers or reproductives depends on the division of labour mechanism of the cells’ lineage founders. Our analytical model focus on two special cases: fully random specialisation and fully coordinated specialisation. We denote $q$ as the target proportion of helpers of the founder of a focal lineage and $Q$ as the target proportion of helpers of all other lineage founders in the group. When the focal lineage employs fully random specialisation, each cell in the lineage may become a helper independently of all other cells in the group and with probability equal to $q$. When the lineage employs fully coordinated specialisation, cells of the lineage interact with other cells from the same lineage to ensure that the proportion of helpers produced by the lineage is exactly equal $q$.

*1.2 The fitness of fully random specialisers*

We can write the fitness of a focal founder in a population where all cells employ random specialisation as:

$w_{FR}\left( q,Q \right)=\sum_{0}^{\left( l-1 \right)m} \binom{\left( l-1 \right)m}{j}Q^{j}{(1-Q)}^{\left( l-1 \right)m-j}\sum_{0}^{m} \binom{m}{i}q^{i}\left( 1-q \right)^{m-i}\left( 1-i/m \right)\left( 1-\epsilon+{\epsilon\left( i+j \right)}/{lm} \right)$ (S1)

where the inner sum iterates over the number of helpers produced in the focal lineage, $i,$ and the outer sum iterates over the number of helpers produced in the other lineages, $j$. In each case, the probability of producing that number of helpers is equal to the associated probability in the binomial distribution. These probability terms can be simplified with new notation: $f\left( j \right)=\binom{\left( l-1 \right)m}{j}Q^{j}{(1-Q)}^{\left( l-1 \right)m-j}; g\left( i \right)=\binom{m}{i}q^{i}\left( 1-q \right)^{m-i}$. We can simplify Equation (S1) by noting that $\sum_{0}^{m} g\left( i \right)i=mq, \text{ }\sum_{0}^{m} g\left( i \right)i^{2}=mq\left( 1-q \right)+m^{2}q^{2}$ and $\sum_{0}^{\left( l-1 \right)m} f\left( j \right)j=\left( l-1 \right)mQ$. This gives Equation (1) in main text:

$w_{FR}(q,Q)=\left( 1-q \right)\left( 1-\epsilon+{\epsilon q}/l+{\epsilon\left( l-1 \right)Q}/l-{\epsilon q}/{lm} \right).$ (S2)

Let us consider another case: the fitness of a fully random founder in a population where all other group founders are fully coordinated specialisers. Because fully coordinated founders produce an exact proportion of helpers, $Q$, there is only one summation in the fitness expression:

$w_{FR}(q,Q)= \sum_{0}^{m} g(i)\left( 1-i/m \right)\left( 1-\epsilon+{\epsilon\left( i+(l-1)mQ \right)}/{lm} \right)$. (S3)

By applying the properties of $\sum_{0}^{m} g\left( i \right)i$ and $\sum_{0}^{m} g\left( i \right)i^{2}$, we get Equation (1) in main text again. This is because the lack of higher order terms of $j$ in Equation (S1), and the first-order summation of random founders, $\sum_{0}^{\left( l-1 \right)m} f\left( j \right)j=\left( l-1 \right)mQ$, gives the same results of fully coordinated founders (the last term of Equation (S3)).

*1.3 Derivation and analysis of the ESS for the target proportion of helpers of fully random specialisers*

Since Equation (S2) is a convex function, we solved $\frac{dw_{FR}\left( q,Q \right)}{dq}\left. \right|_{q=Q=q^{*}}=0$ to find the $q$ where fitness is maximised,

$q_{FR}^{*}=\frac{lm\left( \epsilon-1 \right)+\epsilon\left( m-1 \right)}{\epsilon\left( lm+m-2 \right)}$. (S4)

By taking partial derivatives of Equation (S3), we find that: increasing essentiality (higher $\epsilon),$and smaller lineages sizes (smaller $m)$produce a higher target proportion of helpers (larger $q_{FR}^{*}$).

We show below the derivative of the ESS proportion of helpers with respect to the number of lineages,$l$:

$\frac{\partial\left( q_{FR}^{*} \right)}{\partial l}= \frac{-m\left( \epsilon+m-2 \right)}{{\epsilon\left( lm+m-2 \right)}^{2}}$. (S5)

Given the biological meaningful range of the parameters, $0.5\leq\epsilon\leq1$, $l\geq1$, and $m\geq1$, the denominator is always positive and increase with increasing $l$. Thus, the sign of the first order derivative depends on $\left( \epsilon+m-2 \right)$, which is positive because $m\geq2$ is the necessity condition for division of labour to take place. In other words, whenever division of labour is favoured ($q_{FR}^{*}>0$), a higher relatedness (smaller $l$) leads to a higher target proportion of helpers (larger $q_{FR}^{*}$).

*1.4 The fitness of fully coordinated specialisers*

The fitness of founders taking fully coordinated specialisation is relatively straight-forward, because the exact proportions of helpers can be reached through coordination:

$w_{FC}(q,Q)=(1-\theta)(1-q)(1-\epsilon+{\epsilon q}/l+{\epsilon\left( l-1 \right)Q}/l)$, (S6)

where the first part is the cost of coordination, the second part is the loss from producing helpers, and the third part is the baseline fitness and the total amount of help received from the social group. Note that the fitness of coordinated founder in a population where all other founders take random specialisation is the same, since $\sum_{0}^{m\left( l-1 \right)} f\left( j \right)j/lm$ is equal to the last term of Equation (S6), ${\epsilon\left( l-1 \right)Q}/l$.

*1.5 Derivation and analysis of the ESS for the target proportion of helpers of fully coordinated specialisers*

Similar to the optimal target proportion of helpers of fully random specialisers, the ESS of fully coordinated specialisers can be found by solving $\frac{dw_{FC}(q,Q)}{dq}\left. \right|_{q=Q=q^{*}}=0$ from Equation (S6),

$q_{FC}^{*}=\frac{\epsilon l+\epsilon-l}{\epsilon\left( l+1 \right)}$, (S7)

By taking partial derivatives of Equation (S7), we find the same results as $q_{FR}^{*}$ that: increasing essentiality (higher $\epsilon),$and increasing relatedness (smaller $l)$produce a higher target proportion of helpers (larger $q_{FC}^{*}$). Yet, the target proportion of fully coordinated specialisers is independent with lineage size ($m$).

*1.6 Analysing the difference in ESS target proportions of helpers*

From Equation (2) and (4), we can write the difference in target proportions as $q_{FC}^{*}-q_{FR}^{*}=\left( \epsilon l+\epsilon-l \right)/\left( \epsilon\left( l+1 \right) \right)-\left( lm\left( \epsilon-1 \right)+\epsilon\left( m-1 \right) \right)/\left( \epsilon\left( lm+m-2 \right) \right)$. By assuming $lm+m-2>0$, the condition for division of labour to exist, we can simplify the expression as

$q_{FC}^{*}-q_{FR}^{*}=\frac{2l-\epsilon\left( l+1 \right)}{\left( \epsilon\left( l+1 \right) \right)\left( \epsilon\left( lm+m-2 \right) \right)}$, (S8)

where the denominator is positive, and the numerator is never negative because $\epsilon<1$ and $l$ is at least 1. As a result, the ESS proportion of fully coordinated specialisers is greater or equal to which of fully random specialisers ($q_{FC}^{*}\geq q_{FR}^{*}$). Note that our assumption, $lm+m-2>0$, is required for division of labour to take place, because it is only violated when $l=1$ and $m=1$, where there is only one individual in each social group.

The result that $q_{FC}^{*}\geq q_{FR}^{*}$ helps to explain the existence of places where neither mechanism can invade the other in Fig. 2 of the main text (black regions). The boundaries of successful invasion (blue and orange regions) are described in Expressions (5) and (6) in main text. Considering the case where coordinated mechanism could be selected ($\theta lm<1$), the right-hand side of Expression (5) is smaller than or equal to which of Expression (6) by applying $q_{FC}^{*}\geq q_{FR}^{*}$. Hence, the blue and orange regions in Fig. 2 never overlap.

*1.6 Comparing the costs of fully random mechanism and fully coordinated mechanism*

From the previous derivation, we can rearrange the fitness function of random specialisation as

$w_{FR}(q,Q)= \left( 1-q \right)\left( 1-\epsilon+{\epsilon q}/l+{\epsilon\left( l-1 \right)Q}/l \right)-{\left( \epsilon/l \right)q\left( 1-q \right)}/m$. (S9)

Then, letting $G\left( q, Q \right)=\left( 1-q \right)\left( 1-\epsilon+{\epsilon q}/l+{\epsilon\left( l-1 \right)Q}/l \right)$ and $Var\left( q \right)={q\left( 1-q \right)}/m$, we can simplify the fitness functions,

$w_{FR}(q,Q)=G\left( q, Q \right)-\epsilon/l Var\left( q \right)$. (S10)

Since $\frac{\partial^{2}G\left( q,Q \right)}{\partial q^{2}}= -\frac{2\epsilon}{l}$, we further simplify the fitness function of random specialisation as,

$w_{FR}(q,Q)=G\left( q, Q \right)+\frac{\frac{\partial^{2}G\left( q,Q \right)}{\partial q^{2}}Var\left( q \right)}{2}$ ,

$=\left( 1+\frac{\frac{\partial^{2}G\left( q,Q \right)}{\partial q^{2}}Var\left( q \right)}{2G\left( q, Q \right)} \right)G\left( q,Q \right)$,

$=\left( 1-\varphi\right)G\left( q,Q \right)$, (S11)

where $\varphi=-\frac{\frac{\partial^{2}G\left( q,Q \right)}{\partial q^{2}}Var\left( q \right)}{2G\left( q, Q \right)}$is the cost of random specialisation. By using$G\left( q, Q \right)$, we can also rewrite the fitness of fully coordinated specialisers as,

$w_{FC}\left( q,Q \right)=\left( 1-\theta\right)G\left( q,Q \right)$, (S12)

where the cost of coordination is $\theta$. Note that the two fitness functions may have different optimal $q$. Using (S7) and (S8), we can solve for when a coordinated specialiser can invade a population of random specialisers by evaluating: $w_{FC}\left( q_{FR}^{*},q_{FR}^{*} \right)>w_{FR}\left( q_{FR}^{*},q_{FR}^{*} \right)$, giving the condition:

$\varphi>\theta$, (S13)

which states that the relative cost of random specialisation ($\varphi$) must be larger than the relative cost of coordination ($\theta$).

*1.7 Effects of the cost of coordination on invading conditions*

The effect of the cost of coordination ($\theta$) on the invasion of coordinated specialisers is shown in Fig. S1. When the cost of coordination is larger (higher $\theta$), the parameter spaces where fully coordinated specialisation invades becomes smaller and more sensitive to group size (slope decreased); when the cost of coordination becomes smaller (lower $\theta$), coordinated specialisers can invade in a broader parameter spaces and the boundary is more sensitive to essentiality of cooperation. Yet, qualitatively coordinated specialisation is still favoured when essentiality is high and when groups are small (lower right corners of Fig. S1).


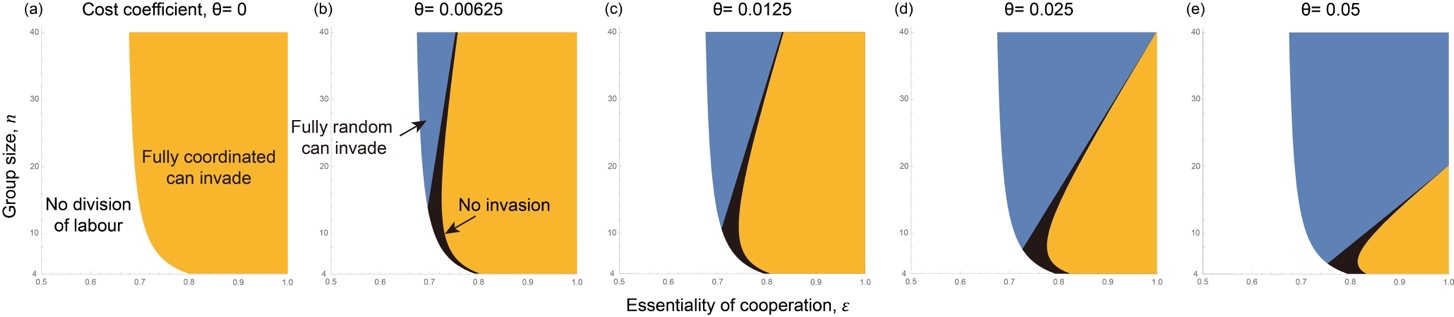


Figure S1. The effects of cost coefficient of coordination ($\theta$) on the invasion conditions in the analytical model, where the blue region is the spaces where random specialisation can invade coordinated specialisation, the orange region is coordinated specialisation invading random, the black region is spaces that neither can invade, and the white region is no division of labour (same as Fig. 2). Each panel shows the conditions of a coefficient value. In the main figures, the cost of coordination is set to $\theta=0.025$ (panel d). Though we only show the analyses of non-clonal groups here, $R=0.5$, the qualitative effect of the cost of coordination is not affected by the relatedness of the population.

**2. A more complicated analytical model: Mutant founders invade with their own optimal target proportions of helpers**

Here, we derive another analytical model where the invading founder does not take the same strategy (i.e., proportion of helpers) as the resident founders. Since the fitness functions remain the same, we only show the derivation of invading conditions. The main result of this model is presented in Fig. 3 of main text.

*2.1 Invasion condition for fully coordinated specialisers invading fully random specialisers*

Assuming the mutant and fully coordinated founder is not taking $q_{FR}^{*}$, we can fund the optimal target proportion of helpers of the mutant founder by solving $\frac{dw_{FC}(q,Q=q_{FR}^{*})}{dq}=0$,

$\hat{q}_{FCInv}=\left( l\left( 2m\left( \epsilon-1 \right)-\epsilon+2 \right)+\epsilon\left( 2m-3 \right) \right)/\left( 2\epsilon\left( lm+m-2 \right) \right)$. (S14)

After substitution, we have

$w_{FC}(\hat{q}_{FCInv},q_{FR}^{*})={{-\left( \epsilon-l\left( \epsilon+2m-2 \right) \right)}^{2}\left( \theta-1 \right)}/{4\epsilon l\left( lm+m-2 \right)^{2}}$, (S15)

and

$w_{FR}\left( q_{FR}^{*},q_{FR}^{*} \right)={\left( m-1 \right)\left( -lm+\epsilon\right)^{2}}/{\epsilon lm\left( lm+m-2 \right)^{2}}$. (S16)

By assuming division of labour occurs, $lm+m>2$ (see section 1.4 for more details), we can simplify the inequality $w_{FC}(\hat{q}_{FCInv},q_{FR}^{*})>w_{FR}\left( q_{FR}^{*},q_{FR}^{*} \right)$ as $\frac{-m\left( \theta-1 \right)\left( \epsilon-l\left( \epsilon+2m-2 \right) \right)^{2}}{4\left( m-1 \right)\left( \epsilon-lm \right)^{2}}>1$. We solve the roots of $\epsilon$,

$\epsilon<\frac{2\left( \sqrt{-l^{2}m\left( m-1 \right)\left( lm+m-2 \right)^{2}\left( \theta-1 \right)}+lm\left( m-1 \right)\left( \theta+1 \right)+l^{2}m\left( m+\theta-\theta m-1 \right) \right)}{m\left( \theta+l\left( l-2 \right)\left( \theta-1 \right)+3 \right)-4}$, (S17.1)

$\epsilon>\frac{2\left( \sqrt{-l^{2}m\left( m-1 \right)\left( lm+m-2 \right)^{2}\left( \theta-1 \right)}-lm\left( m-1 \right)\left( \theta+1 \right)+l^{2}m\left( m+\theta-\theta m-1 \right) \right)}{m\left( \theta+l\left( l-2 \right)\left( \theta-1 \right)+3 \right)-4}$, (S17.2)

where Equation (S17.2) is in biologically meaningful parameter space and plotted in Fig. 3.

*2.2 Invasion condition for fully random specialisers invading fully coordinated specialisers*

Similar with the previous section, we find the optimal strategy of the mutant founder from solving $\frac{dw_{FR}(q,Q=q_{FC}^{*})}{dq}=0$,

$\hat{q}_{FRInv}=\left( 2\epsilon m-\epsilon-l\left( \epsilon-2m\left( \epsilon-1 \right) \right) \right)/\left( 2\epsilon\left( l+1 \right)\left( m-1 \right) \right)$. (S18)

After substituting $w_{FR}\left( \hat{q}_{FRInv},q_{FC}^{*} \right)>w_{FC}\left( q_{FC}^{*},q_{FC}^{*} \right)$, we have $\frac{\left( \epsilon+l\left( \epsilon-2m \right) \right)^{2}}{4l^{2}m\left( m-1 \right)\left( \theta-1 \right)}<1$. Then we find the roots for $\epsilon$,

$\epsilon< \frac{2\left( l^{2}m+lm+\sqrt{-m\left( m-1 \right)\left( l^{2}+l \right)^{2}\left( \theta-1 \right)} \right)}{\left( l+1 \right)^{2}}$, (S19.1)

$\epsilon> \frac{2\left( l^{2}m+lm-\sqrt{-m\left( m-1 \right)\left( l^{2}+l \right)^{2}\left( \theta-1 \right)} \right)}{\left( l+1 \right)^{2}}$. (S19.2)

where Equation (S19.1) is in biologically meaningful parameter space and plotted in Fig. 3.

**3. Additional simulations**

We run a series of supplementary simulations to help understand the behaviour of simulations. In section 3.1, we examine the proportion of helpers without the presence of coordination and show it is very close to analytical results. Section 3.2 shows the difference in analytical and simulative results is resulted from interactions between coevolving traits. Section 3.3 tests the robustness of simulations by initiating with different proportions of helpers. Section 3.4 uses the same assumption as the analytical model that coordination only operates within the same lineage. We explain why coordination is less favoured in these simulations. In section 3.5, we test an alternative coordination cost design: linear cost and find the results remain qualitatively the same. Section 3.6 explains some unexpected patterns of section 3.5. The biological details of simulation are described in main text, we also provide pseudocode for the simulations run in the main text in section 3.7.

*3.1 Evolved proportion of helpers under fully random specialisation*

As additional verification between the analytical model and the simulations, we ran simulations where no coordination takes place. In this single-trait evolving scenario, we found the evolved proportion of helpers ($\bar{P}_{Rand}$) is almost identical with the ESS target proportion of helpers ($q_{FR}^{*}$) in the analytical model (Fig. S2e-h and Fig. 2i-l).


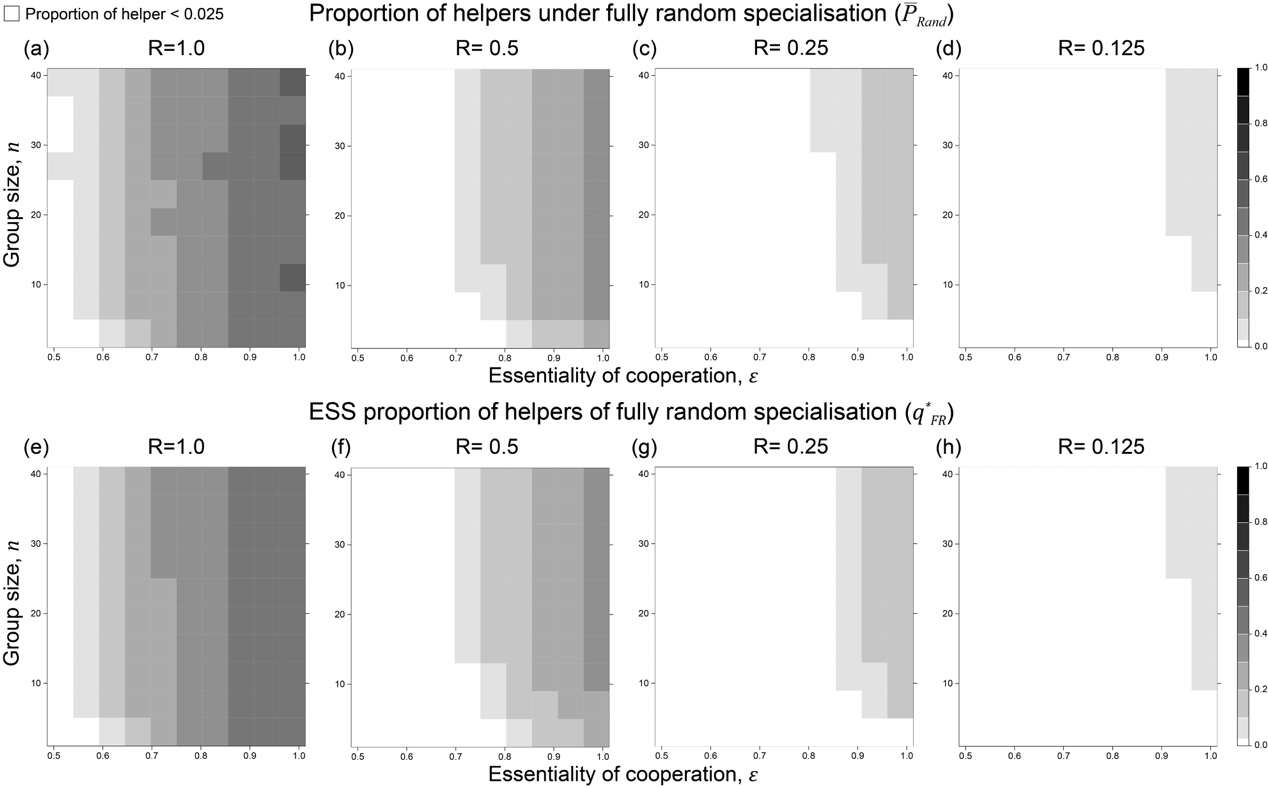


Figure S2. The optimal proportion of helper, or level of cooperation, of fully random specialisers in simulations and the analytical model. (a-d) We run single-trait evolving simulation where only target proportions of helpers are evolving, while coordination level is fixed at 0. Darker shades mean there are more helpers in the steady-state populations. All data are extracted in the last 10% of 10^5^ generation simulations with 10 repetitions for each parameter combination and group size is equal to number of lineages times lineage sizes ($n=lm$). (e-h) The corresponding proportion of helpers in analytical model. We plot the same $q_{FR}^{*}$ as in Fig. 2i-l but in identical way as simulations for better comparison.

*3.2 Interactions between coevolving traits explain some discrepancies between simulations and analytical results in non-clonal groups*

In main text we found the analytical model and simulations have the same qualitative results on optimal mechanisms when groups are clonal (Fig. 2a, Fig. 5a), but there are some disagreements when relatedness is 0.5 (Fig. 2b; Fig. 5b). These differences can arise from potential interactions between the coevolving helper proportion and the level of coordination, because one trait controls the average number of helper whereas the other trait controls the variation in number across groups. Thus, we run a set of simulations with the proportion of helpers fixed at the ESS of fully random specialisers ($q_{FR}^{*}$), and only evolving levels of coordination.

The single-trait simulations have very similar pattern to which in analytical models (Fig. S3b; Fig. 2b; Fig, 3b). In particular, high levels of coordination ($\bar{S}$) are only evolved in small groups ($n$) (Fig. S3b), whereas the coevolved simulations show high coordination when essentiality ($\epsilon$) is high, regardless of group size (Fig. S3a). In addition, we also found the proportion of helpers ($\bar{P}$) in single-trait simulations are higher than which in coevolving simulations (Fig. S3c-d). Altogether, these results suggest interactions between coevolving traits are likely to be the cause of different patterns.


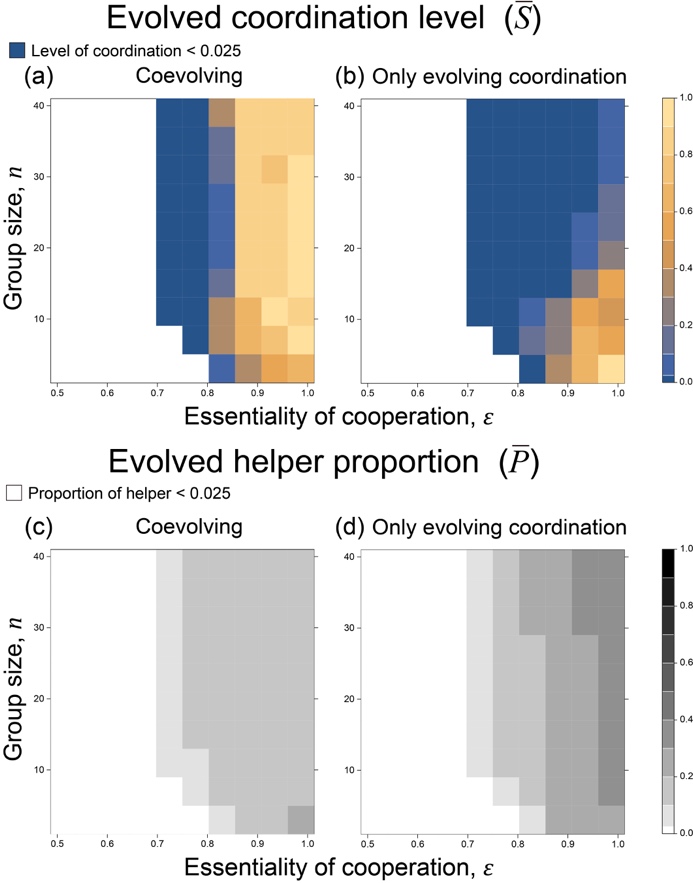


Figure S3. Comparison of coevolving and single-trait-evolving simulations in non-clonal population (R= 0.5). The first row is the coevolved or evolved level of coordination (a-b) and the second row is the coevolved or phenotypically expressed proportion of helpers (c-d). Importantly, the right column shows the result where target proportion of helper ($q$) is set at the ESS of random specialisers ($q= q_{FR}^{*}$) and helper proportion is not evolving. On the other hand, panel a and c are identical with panel b and f of Fig. 5, where both coordination and proportion are evolving. Similar to Fig. 5, coordination levels with proportion of helper less than 0.025 are shown in white as we regard it as no division of labour being favoured and group size, $n$, is equal to number of lineages ($l$) times lineage size ($m$).

*3.3 Sensitivity analysis of initial target proportion of helpers*

Simulations of various initial target proportions of helpers ($q$) all show very similar patterns in both coevolved proportions of helpers as well as coevolved coordination levels (Fig. S4). These results suggest the initial proportions do not change the outcome of coevolving traits.


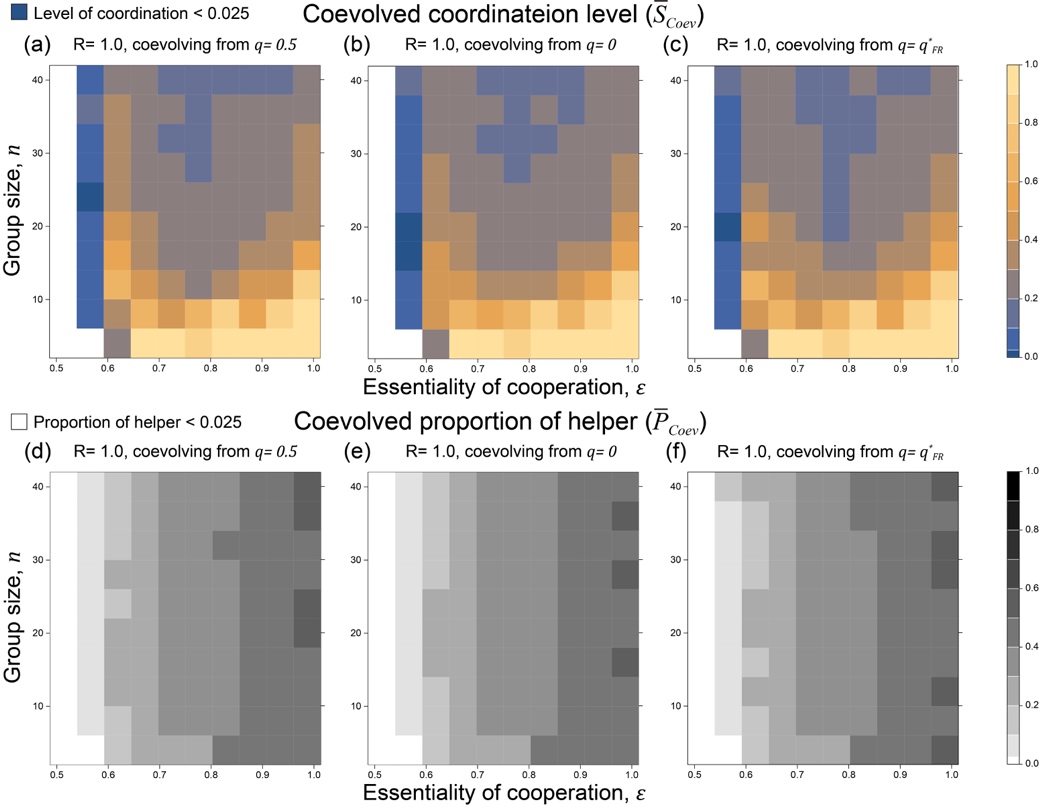


Figure S4. The coevolved coordination level and proportion of helpers under various initial states in clonal population (R=1). Simulations are initiated with target proportion of 0.5 (a), 0 (b), and ESS proportions of fully random specialisers (c). All coevolving simulations in the main text and supplementary information have the initial proportion of helpers is 0.5 if not stated otherwise.

*3.4 Simulations where individuals only coordinate with clonemates*

Let us consider a simulation closer to our analytical model: group members can only be coordinated with individuals of the same lineage (from the same founder). The simulations show both coordination level ($\bar{S}_{coev}$) and proportion of helpers ($\bar{P}_{coev}$) are lower (Fig. S5) comparing to the cases where individuals can be coordinated with the entire group (Fig. 5).

This difference happens because the analytical model assumes group size is large and coordinated specialisation can reach exact optimal proportion of helpers. However, the simulations the available proportion of helpers becomes more discrete as group size becomes smaller. That is, for a given group size $n$, there are only $(n+1)$ achievable proportions of helpers ($x/n;\left\{ x\mathbb{\in N} \right| 0\leq x\leq n)$). If the group size is held constant, the minimal non-zero coordinated proportion of helpers increases ($l/n$) with decreasing relatedness. Thus, given that the ESS proportion of helpers is known to decrease with decreasing relatedness (section 1.6) whereas the minimal possible level of coordination is increasing, coordinated specialisation is less favoured because desired proportion cannot be reached through coordination.


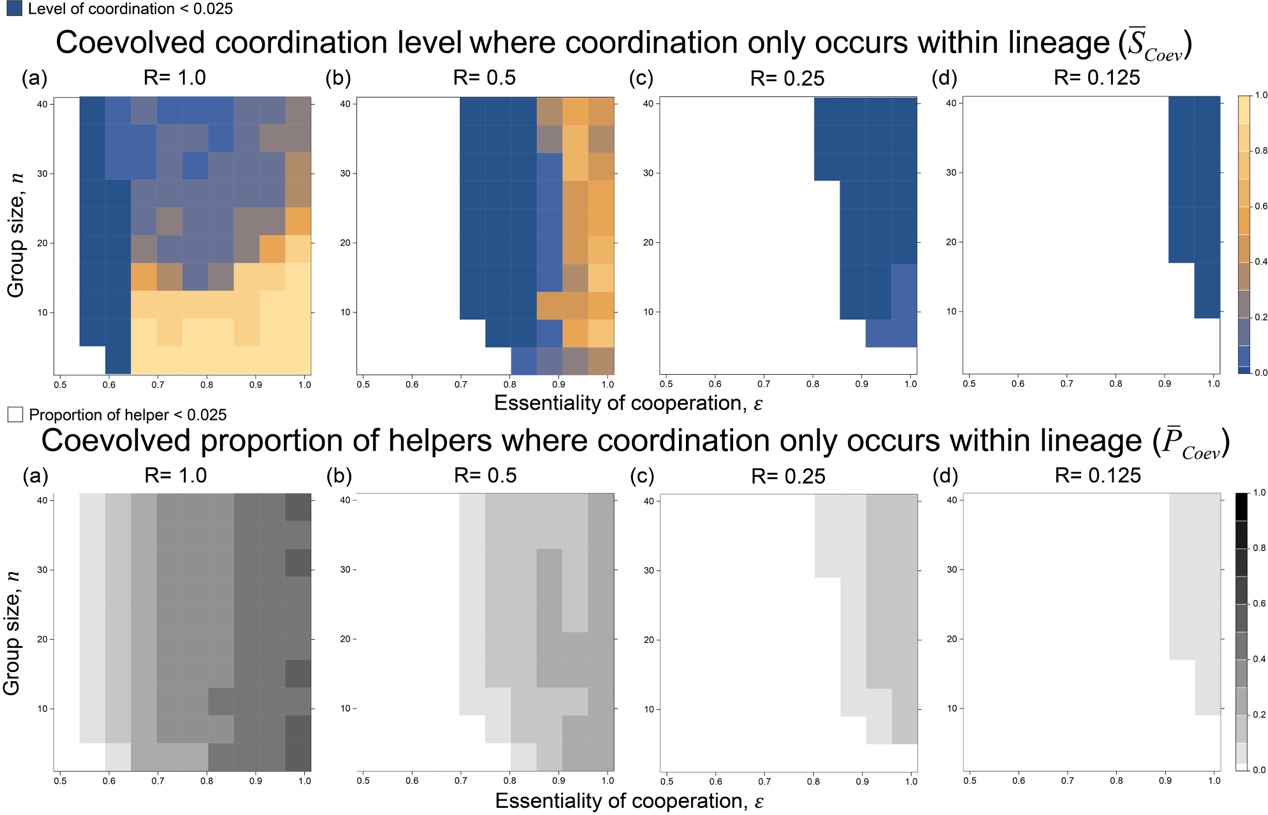


Figure S5. The coevolved coordination level and proportion of helpers in the simulations when coordination only takes place within lineage, which contains the individuals originated from the same founder in the social group. (a-d) The evolved coordination level plotted against the essentiality of cooperation (x-axis) and group size (y-axis). Each panel represents the results of different relatedness setting. The blueish colours represent the evolved mechanism of division of labour is closer to random specialisation, whereas the orangish colours represent the evolved mechanism is closer to coordinated specialisation. Each grid is an average of 10 repeated simulations where the average is taken from the last 10% of 10^5^ generations. (e-h) The evolved proportion of helpers. Darker shades mean there are more helpers in the population. Coordination levels when proportion of helpers is below 0.025 is not plotted as we regard it as no division of labour.

*3.5 Linear coordination cost*

Here, we apply a simpler cost design where the cost of coordination increases proportionally with coordination level, $cost\left( s \right)=\theta s$. In terms of the effects of relatedness, the results (Fig. S6) are generally similar with which of decelerating coordination costs (Fig. 5). Yet, the linear cost simulations have more gradual transitions of coordination levels (Fig. 5a and S6a). In addition, proportion of helpers is lower in the linear cost simulations (Fig. 5e-h and S6e-h).


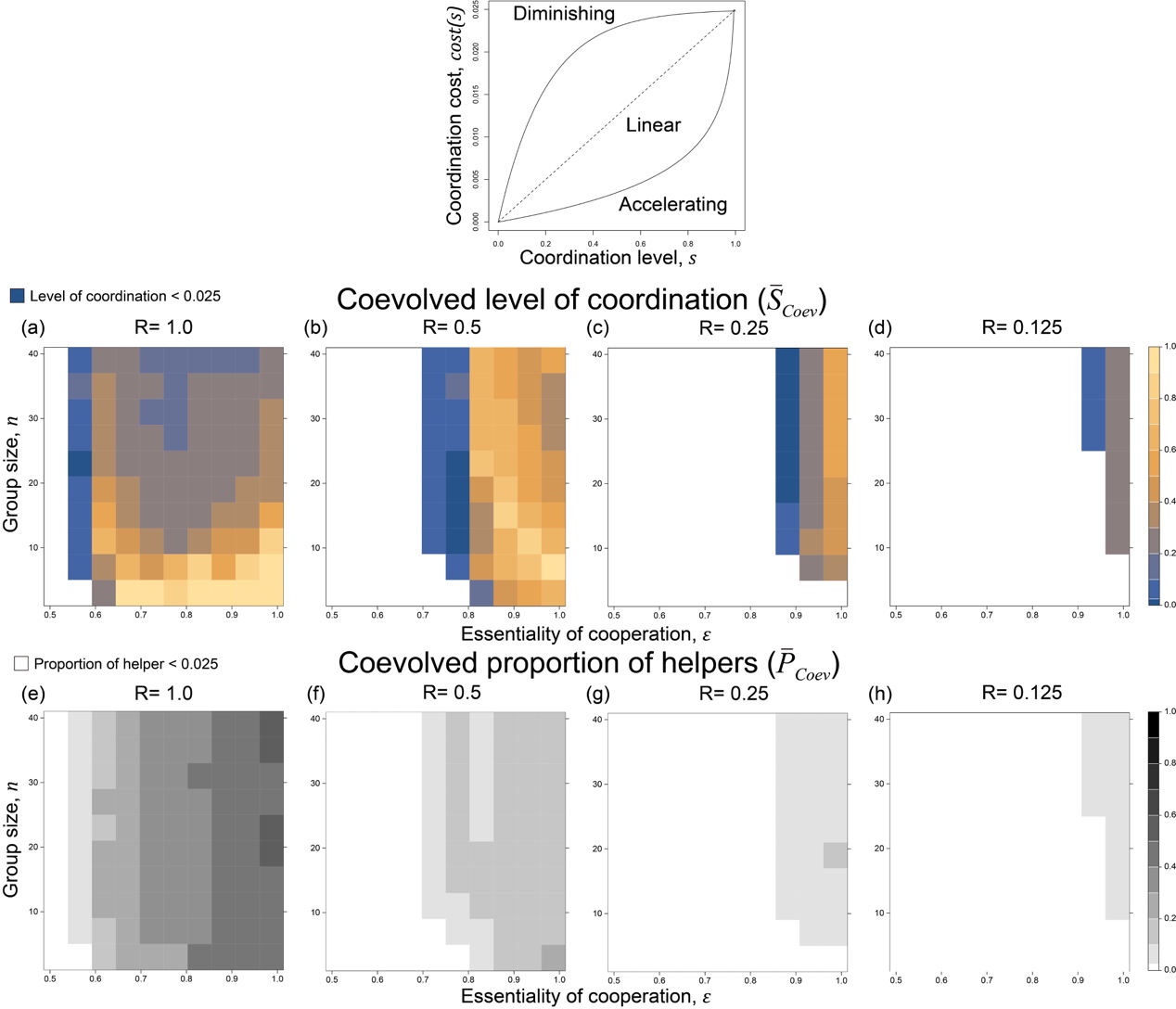


Figure S6. The coevolved level of coordination and proportion of helpers in the simulations when coordination cost is linear. The top panel shows the comparison of cost functions for coordination, where the linear cost is used in SI section 3.5-3.6; the accelerating cost is used in SI section 3.7; and the decelerating cost is used in all other simulations.

*3.6 Single trait evolution versus two traits coevolution under linear cost*

Despite the similarity, there are some qualitative discrepancies between the two simulations of linear and decelerating coordination cost. That is, we found high levels of coordination ($\bar{S}$) when essentiality ($\epsilon$) is around 0.6 in clonal groups (Fig. S7a; identical to Fig. S6a), which is not found in simulations with decelerating cost of coordination (Fig. 5a).

Similar to section 3.2, we suspected this unexpected pattern arises because of the potential interactions between the coevolving traits and ran another set of simulation where proportion of helpers is not evolving. Since we are interested in the cases where coordinated specialisation evolving from random specialisation, we fix the target proportion of helpers at the ESS proportion of random specialisers (Fig. S2) and let coordination level be the only evolving trait. The single-trait simulations show similar pattern to coevolving simulations with decelerating cost of coordination (Fig. S7b; Fig. 5a). On the other hand, there is no big difference in proportion of helpers between single-trait and coevolving simulations (Fig. S7c-d). This is because proportion of helpers has a larger impact on the individual fitness and thus is less likely to deviate from analytical results (Equation (10) and Fig. 4). All in all, our results of evolving coordination simulations suggest interactions between traits are likely to be the cause of the unexpected pattern.


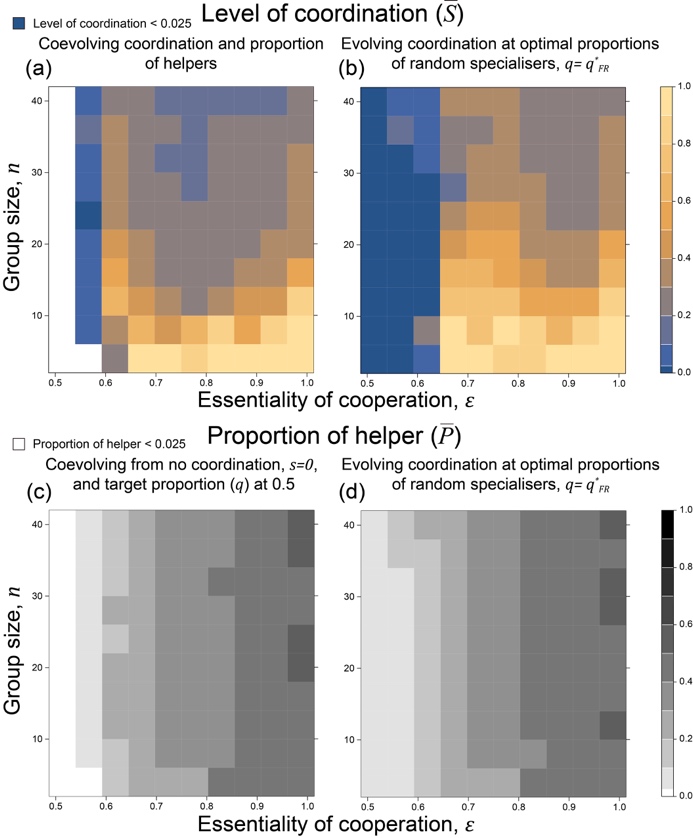


Figure S7. Comparison of dynamics between coevolving traits and single-evolving trait when the cost of coordination is linear. Similar to Fig. S3, the first row is the level of coordination (a-b) and the second row is the proportion of helpers (c-d). Importantly, the right column shows the result where target proportion of helper ($q$) is set at the optimum of random specialisers ($q= \bar{q}_{rand}$; Fig. S2a): evolved level of coordination is shown in panel b and the phenotypic proportion of helper from fixed target proportion is shown in panel d. On the other hand, panel a and c are identical with panel a and e in Fig. S6, where both coordination and proportion are evolving.

*3.7 Single trait evolution versus two traits coevolution under accelerating cost*

When the cost for coordination is accelerating, it implies low levels of coordination would have a very similar cost to no coordination and reaching perfect coordination is very costly. As a result, we see coordination level is higher than which in linear or decelerating cost in general, but regions with full coordination is fewer (Fig. S8; in comparison with Fig. S3 and S7). Nevertheless, the parameter spaces with small group size and high essentiality still have greatest level of coordination (group size of 4 and essentiality of 1). In addition, we found interactions between coordination and target proportion of helpers affect the evolved level of coordination, and the unexpected pattern disappears when only coordination is evolving (Fig. S8a-b).


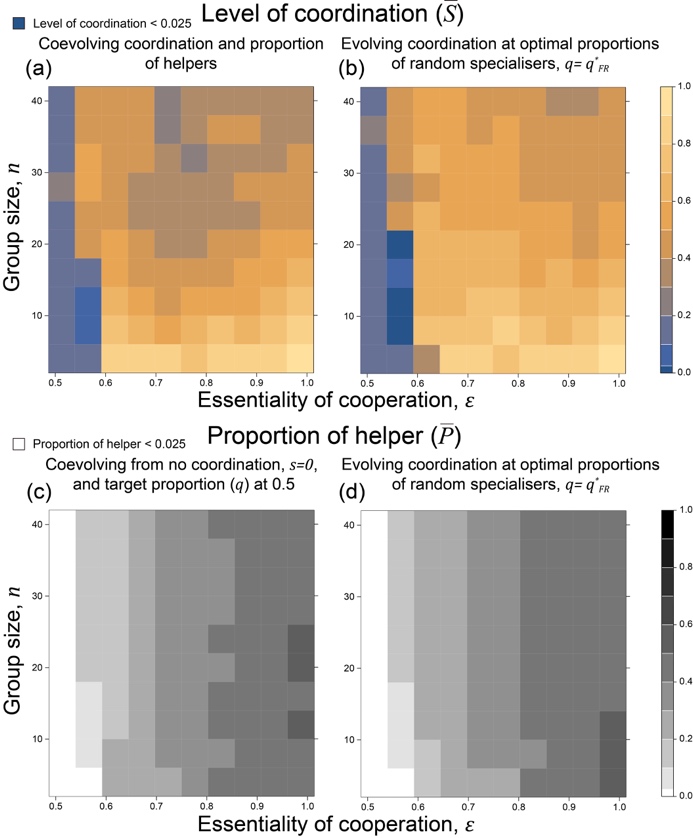


Figure S8. Comparison of dynamics between coevolving traits and single-evolving trait when the cost of coordination is accelerating. See Fig. S3 for plotting details.

*3.8 Pseudocodes of the simulations*

Pseudocode is provided below, please check data availability section of main text for the link to source codes.

// Initialisation

Define the essentiality of cooperation ($\epsilon$), number of lineages ($l$), group size ($lm$), cost coefficient of coordination ($\theta$), mutation rate ($p_{mut}$), and the distribution of the deviations of mutated traits;

Population size= $lm\left\lceil{{10}^{4}}/{lm} \right\rceil$; Number of groups= $\left\lceil{{10}^{4}}/{lm} \right\rceil$;

Create the population matrix [number of groups, group size, list of individual properties];

Create the coordination matrix [group size, group size];

Create the list for storing fitness [population size];

Initialise each individual with desired values in the list of individual properties: target proportion of helper ($q$), coordination level ($s$), and division type ($h$). Initial population has uniform trait values;

// Main iteration

For each generation (1 till 10^5^) {

For each group (1 till number of groups) {

// Coordination

For each individual (1 till group size) {

Determine whether other individuals are coordinated with focal individual, probability is the coordination level of focal individual;

}

// Dividing labour

For number of sampling (1 till 5* group size) {

Sample one individual from the group;

Observe the developmental plans of coordinated group members of the focal individual and get its observed proportion of ‘intended helper’;

If (observed proportion is lower than target) change developmental plan to helper;

If (observed proportion is higher than target) change developmental plan to reproductive;

}

// Fitness calculation

Get the number of helpers of the group;

Calculate the fitness of each individual in the focal group;

}

// Reproduction and mutation

For each group (1 till number of groups) {

For number of founders {

Sample one individual from current population, probability is weighted by relative fitness;

If (mutation of a trait, target proportion of helpers or level of coordination, occurs){

Sample the size of mutation from the distribution;

Change the trait value;

Boundary check and fix values at 0 or 1 if exceeded;

}

Paste trait value to the future founder;

}

// Initialising the next population

Create the future group of individuals from the founders: if there is no remainder for group size divided by the number of founders, each proportion of individuals inherit the traits from one founder. If there is a remainder, the remainders’ traits are obtained from randomly sampled founders;

}

// Recording

Calculate the average trait values and phenotype values of the population;

// Replacing the population

Overwrite the population matrix with the created population from reproduction section;

}

Clear memory;

End of simulation;
